# Supplementary material for: Coagulative Nucleation in the Copolymerization of Methyl Methacrylate–Butyl Acrylate under Monomer-Starved Conditions
Source: Polymers (Basel). 2023 Mar 24;15(7):1628. doi: 10.3390/polym15071628 (PMC10096953; doi:10.3390/polym15071628)
Supplement: Supplementary file 1 [file polymers-15-01628-s001.zip › polymers-2294544-supplementary.pdf]

Figure S1 shows the kinetics parameters such as instantaneous conversion, global conversion, residual monomer, and R_p/R_a proportion, obtained by the individual polymerization of monomers at a specific addition rate of 2 gmin^{-1} and with the presence of 1.0 wt.% of surfactant. Furthermore, Figure S1 shows a kinetics comparison between the kinetics parameters obtained by the individual polymerization of monomers at a specific addition rate of 2 g min^{-1} and with the presence of 1.0 wt.% of surfactant and the copolymer kinetic parameters labeled in **Figure S2** as the blue triangle (▲) and Batch copolymer with 1.0 wt.% of surfactant marked in Figure S2 with the green inverted triangle (▼).

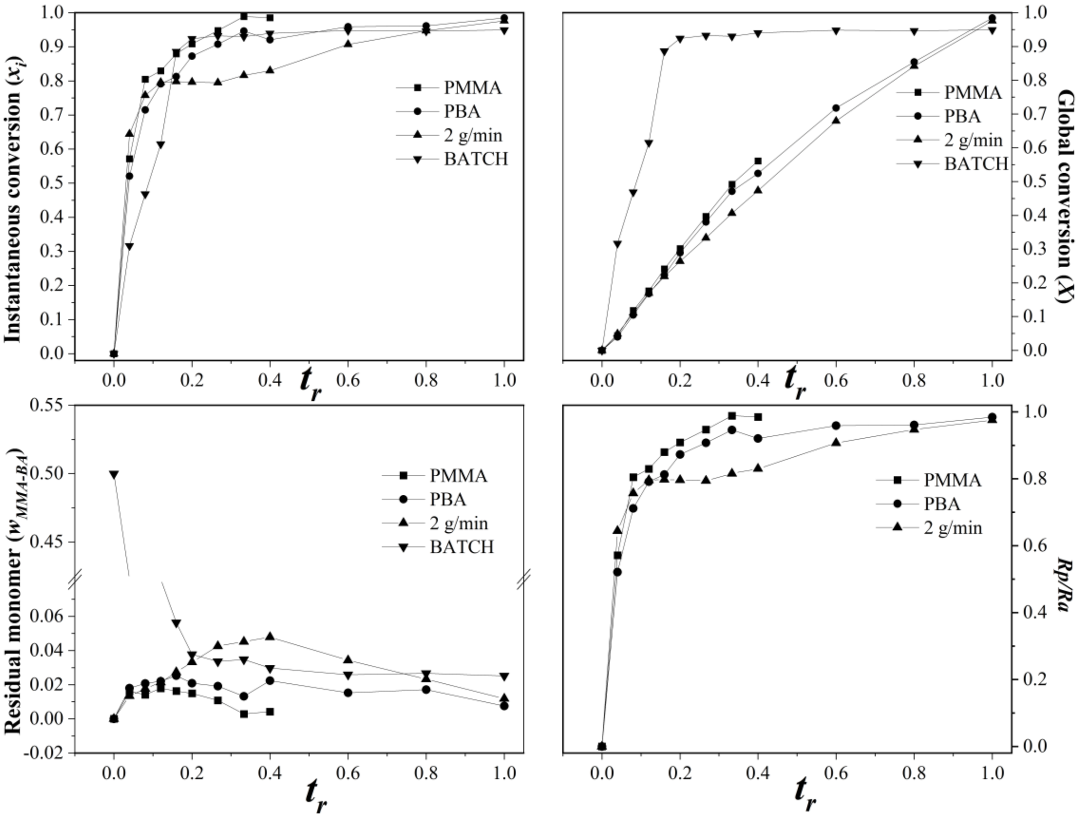

Figure S1. Kinetics parameters of PMMA and PBA homopolymers compared with the copolymer prepared at 2 g min^{-1} and the Batch copolymer with 1.0 wt % of surfactant content.

Figure S2 Shows the comparison between homopolymers and copolymers of particle size and number of particle size calculations.

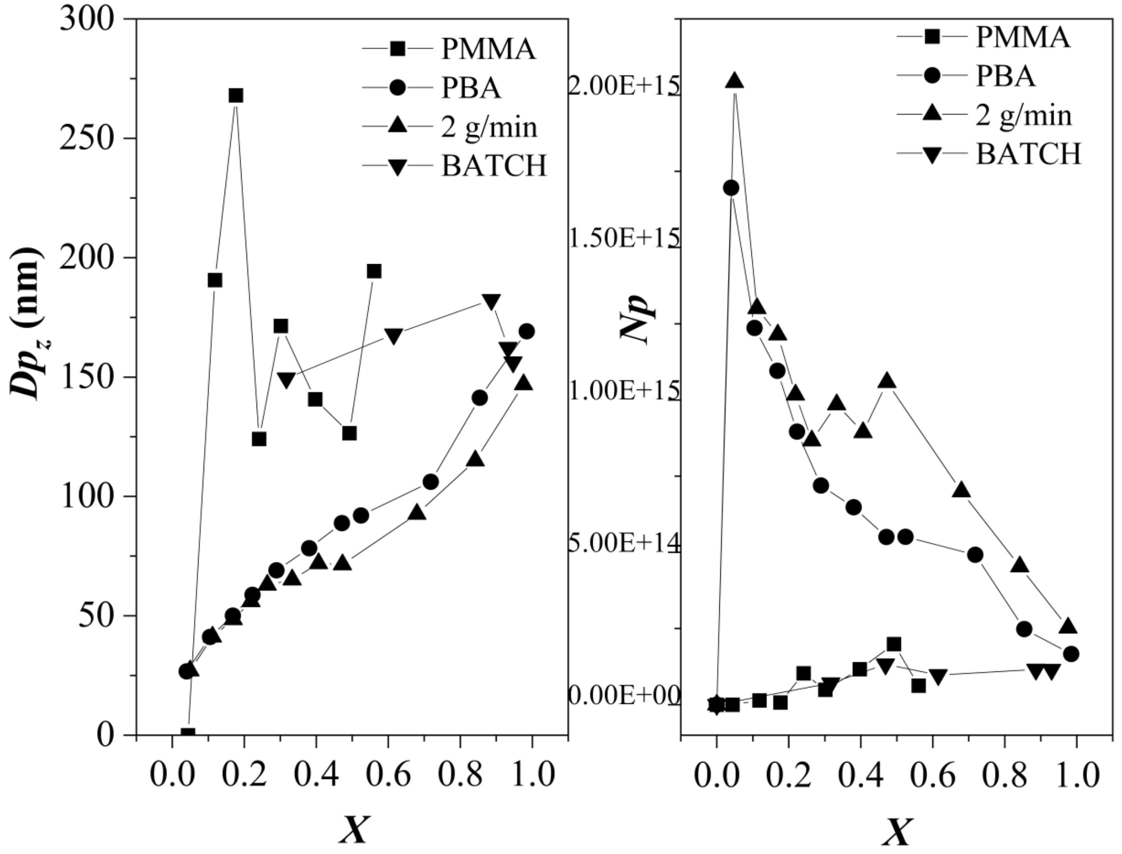

Figure S2. Particle size (Dp_z) and number of particles (N_p) of PMMA and PBA homopolymers compared with the copolymer prepared at 2 g min^{-1} and the Batch copolymer with 1.0 wt.% of surfactant content.

Figure S3 shows the comparison between homopolymer and copolymer surface coverage (S_c) calculations.

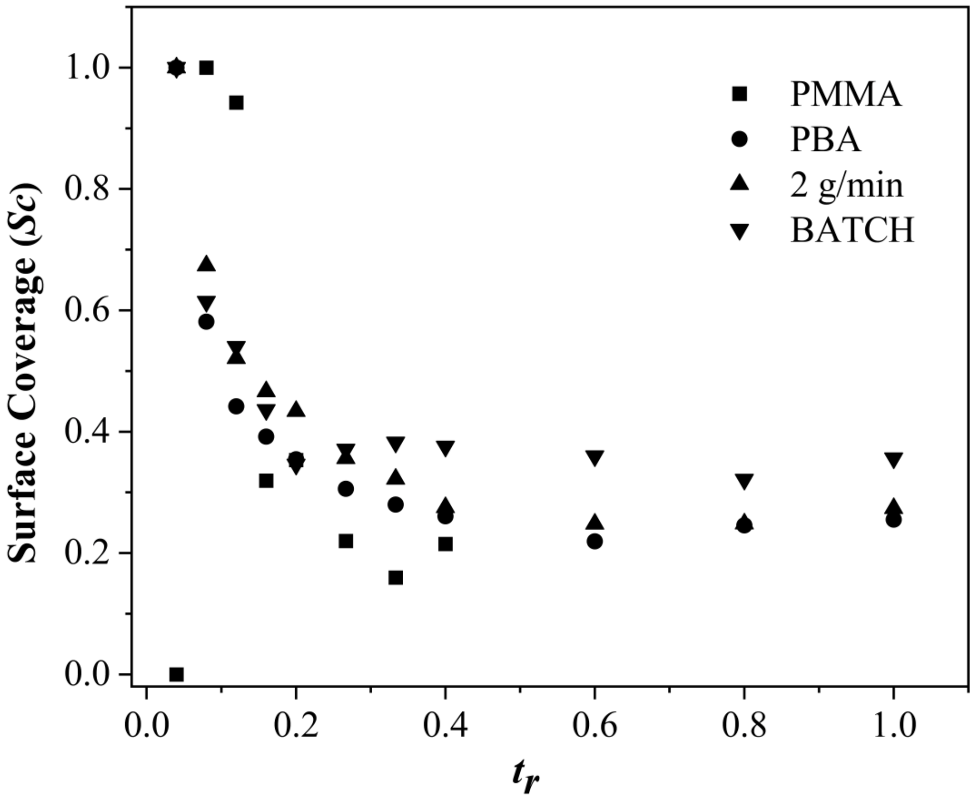

Figure S3. Surface coverage calculations of PMMA and PBA homopolymers compared with the copolymer prepared at 2 g min^{-1} and the Batch copolymer with 1.0 wt % of surfactant content.

Tg calculation according to calorimetric profiles of the copolymers synthesized with comonomers addition rate modification is shown in **Table S1**.

Table S1. Tg of copolymers with 1.0 wt % and 2.0 wt % of surfactant content at different addition rates.

Addition rate	SDS 1.0 wt %	SDS 2.0 wt %
0.5 g/min	21.4 °C	23.1 °C
1.0 g/min	22.3 °C	22.7 °C
2.0 g/min	21.7 °C	21.1 °C
BATCH	20.9 °C	19.6 °C

Figure S4 shows measurements of the Z-potential of selected copolymer samples synthesized with 1.0 wt % of SDS, for the BEP and 2 $gmin^{-1}$ comonomer feed for the SEHP process.

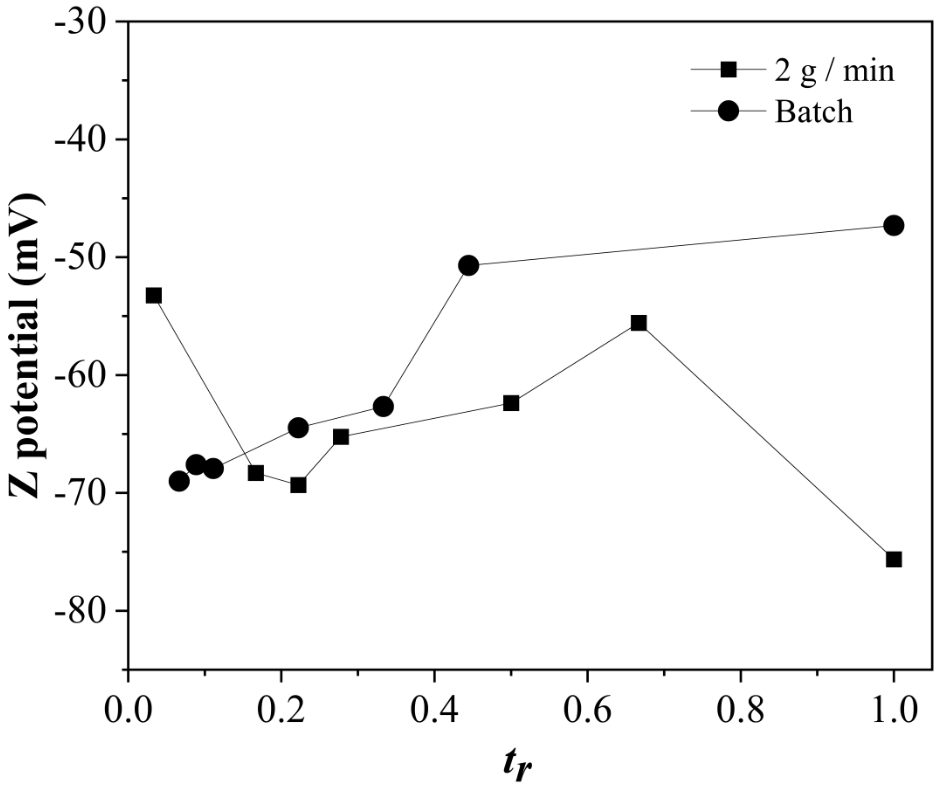

Figure S4. Z-potential measurements for BEP and SEHP process at the feed rate of 2 g/min with 1 wt % of SDS in both processes.

Table S2 shows a resume of stereoregularity of copolymer and BA concentration obtained in selected samples.

Table S2. Tacticity and BA concentration of copolymer obtained by RMN.

**Sample**	**C_{BA} (%)**	**rr (%)**	**rm (%)**	**mm (%)**
0.5 $gmin^{-1}$1SDS	21	63.2	29.2	7.6
0.5 $gmin^{-1}$ 2SDS	19.5	65.1	26.7	8.1
BATCH 1SDS	20	63.3	33.60	3.1

A TEM image of Batch copolymer nanoparticles with 1.0 wt. % of surfactant is shown in Figure S5; homogeneous quasi-spherical shapes were obtained.

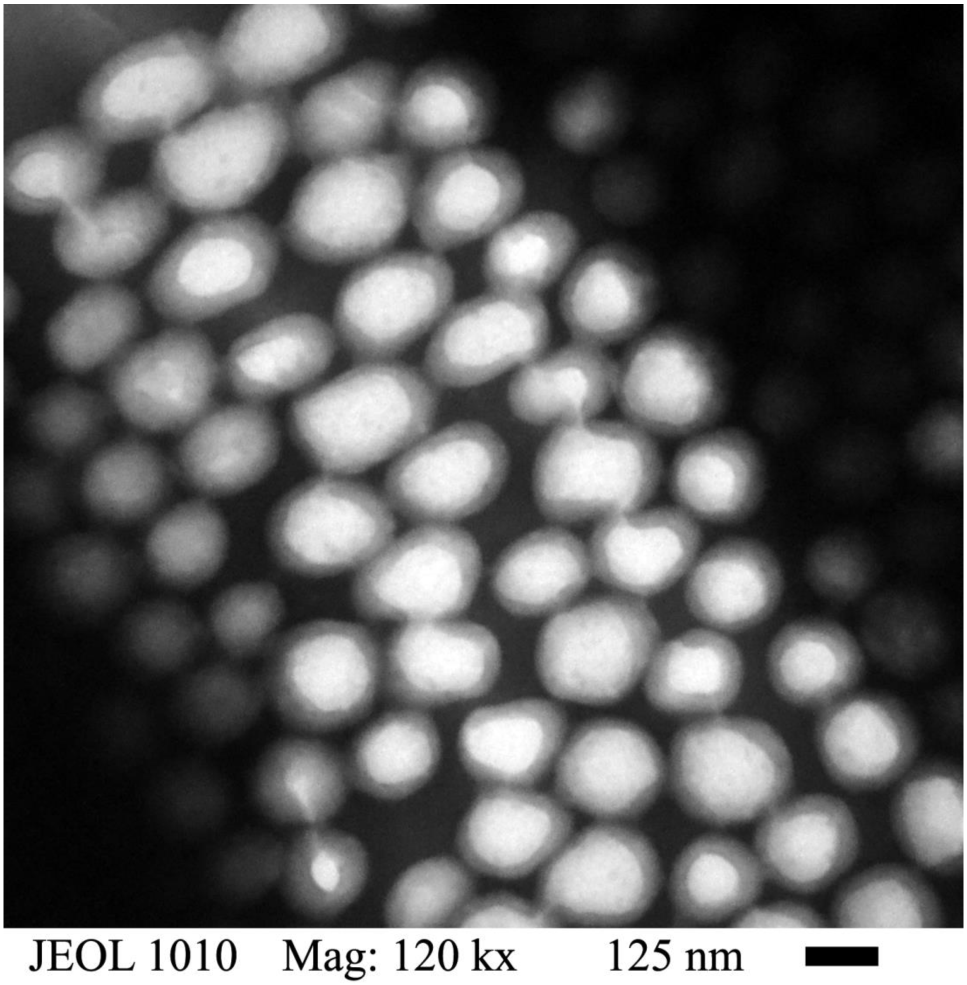

Figure S5. TEM image of final copolymer synthesized with 1.0 wt.% of surfactant content in Batch copolymerization.
